# Habitat suitability of four threatened Himalayan species: Asiatic black bear, common leopard, musk deer, and snow leopard

**DOI:** 10.7717/peerj.16085

**Published:** 2023-09-25

**Authors:** Rajesh Malla, Saroj Panthi, Hari Adhikari, Shiva Pariyar, Rishi Baral, Rukmagat Subedi, Bishnu Prasad Adhikari, Mahesh Poudel, Nischal Sedhai, Megharaj Poudel

**Affiliations:** 1 Forest Research and Training Center, Pokhara, Nepal; 2 Ministry of Industry, Tourism, Forests and Environment, Gandaki Province, Pokhara, Nepal; 3 Department of Geosciences and Geography, University of Helsinki, Helsinki, Finland; 4 National Trust for Nature Conservation, Annapurna Conservation Area, Pokhara, Nepal; 5 Division Forest Office, Mustang, Mustang, Nepal; 6 Division Forest Office, Manang, Manang, Nepal; 7 Division Forest Office, Gorkha, Gorkha, Nepal; 8 Institute of Forestry, Pokhara, Nepal; 9 Division Forest Office, Nawalpur, Nawalparsi (Bardaghat Susta East), Kawasoti, Nepal

**Keywords:** Nepal, Gandaki province, Ecology, Anthropogenic impacts, MaxEnt, Distribution

## Abstract

**Background:**

Biodiversity conservation is becoming challenging day by day. For this, it is essential to understand the distribution, habitat, and impact of anthropogenic activities on animals at risk. We assessed the suitable habitats and anthropogenic impacts on Asiatic black bears, common leopards, musk deer, and snow leopards in and outside the protected areas of Gandaki Province, Nepal.

**Methods:**

We collected the presence locations of Asiatic black bears, common leopards, musk deer, and snow leopards based on scats and other signs. We employed the Maximum Entropy (MaxEnt) tool to identify suitable habitats of our studied species and their anthropogenic impacts on them.

**Results:**

The total suitable habitat of the common leopard was found to be 6,052 km^2^, followed by the Asiatic black bear (5,819 km^2^), snow leopard (4,447 km^2^), and musk deer (1,690 km^2^) in Gandaki Province. Most of the areas of suitable habitat for common leopards and Asiatic black bears were outside the protected areas, and for musk deer and snow leopards were inside the protected areas. Elevation was the most important variable determining habitat suitability of Asiatic black bear, common leopard, and musk deer, whereas the distance to water was the most important variable determining habitat suitability of snow leopard. Asiatic black bears, common leopards, and musk deer face significant anthropogenic impacts, but snow leopards face some anthropogenic impacts.

**Conclusion:**

Managing these animals’ habitats inside and outside protected areas is essential. Hence, biodiversity conservation and livelihood opportunities should be balanced in the Himalayas on a win-win basis.

## Introduction

Conservation of threatened species requires accurate knowledge of their primary attributes, such as distributions and habitats, so conservationists and managers can delineate and optimize management on a priority basis (Lu et al., 2012). For this, determining distributions is crucial for the long-term survival of threatened species in the face of increasing anthropogenic pressures on natural areas (Ebrahimi, Farashi & Rashki, 2017). This includes habitat mapping in the surrounding environment of species where wild animals can accomplish their life cycle (Cody, 1985; Jiang et al., 2012). Nepal started formally conserving biodiversity by establishing the Chitwan National Park in 1973 (Department of National Parks and Wildlife Conservation of Nepal (DNPWC), 2017). After that, Nepal established 20 protected areas to conserve the biodiversity of all geographical regions (Department of National Parks and Wildlife Conservation of Nepal (DNPWC), 2017). Human-wildlife conflicts and anthropogenic impact on wildlife were recorded in some parts of Nepal (Acharya et al., 2016; Panthi et al., 2017). In this scenario, identifying suitable habitats and anthropogenic impact is imperative to conserve the threatened wildlife in Nepal. Based on the data availability, we identified the proper habitat of the Asiatic black bear (*Ursus thibetanus*), common leopard (*Panthera pardus)*, musk deer (*Moschus leucogaster*) and snow leopard (*Uncia uncia*), which are protected by National Parks and Wildlife Conservation Act, 1973 Nepal (GoN, 1973). These species are found in Nepal’s mountain ecosystems, and their survival may ensure the entire ecosystem’s well-being (Lamsal et al., 2018; Panthi, Aryal & Coogan, 2019; Adhikari et al., 2020; Karki & Panthi, 2021).

Among Asiatic black bear, common leopard, musk deer and snow leopard, the Asiatic black bear is found in southern Asia, northeastern China, far eastern Russia, and Japan (Servheen, 1990). The westernmost range limit of this bear is southeastern Iran (Ghadirian et al., 2017). In the Himalayan region of Nepal, the habitat of this species overlaps with that of the red panda (*Ailurus fulgens*) (Bista, Panthi & Weiskopf, 2018). Its prime food species in Nepal is Himalayan bamboo *(Arundinaria* spp) (Panthi, Aryal & Coogan, 2019). The bear is listed as “vulnerable” in the lists of the International Union for Conservation of Nature (IUCN) (Garshelis & Steinmetz, 2016) and included in Appendix I of the Convention on International Trade in Endangered Species of Wild Fauna and Flora (CITES) (CITES, 2019). It has been categorized as an endangered species because of its long-standing human-wildlife conflicts (Ahmadzadeh et al., 2008; Charoo, Sharma & Sathyakumar, 2011; Jnawali et al., 2011; Jamtsho & Wangchuk, 2016; Ghadirian et al., 2017). The habitat of the Asiatic blackbear is declining due to urban and sub-urban encroachment (Escobar, Awan & Qiao, 2015). Crops such as maize (*Zea mays*) and millet (*Paspalum scrobiculatum*) were identified in the diet of this bear in Nepal (Panthi, Aryal & Coogan, 2019). This evidence proves that this is a conflict-causing bear in mountain regions of Nepal.

Common leopards live in various habitats ranging from deserts to forests to high mountains in Asia, Africa, and East Europe (Stein et al., 2020). The Wild Cat Status Survey (IUCN/SSC Cat Specialist Group) has categorized leopards as a vulnerable species (Stein et al., 2020). Due to the high risk of extinction by a trade of its parts, it is included in Appendix I of CITES (CITES, 2019). Wild ungulates cover the primary dietary composition of the common leopard, whereas livestock is also identified as a diet of this leopard in a small amount (Aryal & Kreigenhofer, 2009). The common leopard in Nepal has a conservation focus, with the population estimated at <1,000 individuals. This leopard is a significant conflict-causing wildlife in Nepal (Acharya et al., 2016; Adhikari et al., 2020). Similarly, the common leopard is at high mortality risk due to human-wildlife conflict in central Nepal (Adhikari et al., 2022). Habitat encroachment is a major reason for livestock depredation by this leopard (Maharjan et al., 2017).

Musk deer is a widely but discontinuously distributed flagship species throughout the Himalayas from 3,000 to 4,400 m (Green, 1985, 1986; Shrestha, 1997). In Nepal, it is recorded from 2,300 to 4,300 m in forests dominated by birch and rhododendron (Shrestha, 1998). This species is found in mountainous regions of Nepal, covering 30,177.19 km^2^, of which 5,815.08 km^2^ are located inside protected areas (Aryal & Subedi, 2011). Despite the more potential habitat outside the protected areas, its specific management activities were lacking (Aryal & Subedi, 2011). This lack assisted in a surge in persistent population decline (Aryal et al., 2010). Because of anthropogenic activities such as over-exploitation of forest resources, habitat shrinkage, destruction, and degradation, this species is listed as endangered on the IUCN red list (Timmins & Duckworth, 2015). Also, it has been listed in CITES Appendix I (CITES, 2019) and is protected under the National Park and Wildlife Conservation Act of Nepal. Musk deer dwell in steep, forested, or shrub-covered slopes, mainly in the sub-alpine zones of mountain regions. Dense undergrowth of rhododendron (*Rhododendron anthopogon, R. arboreum*) bamboo (*Arundinaria* spp), and other shrubs constitute the typical habitat (Green, 1987). Habitat use depends on the availability of food, cover, and other factors. They are very shy and solitary animals and may not become active until dusk.

The endangered snow leopard inhabiting the rugged and fragile landscape of the Himalayas (Jackson & Ahlborn, 1989) is one of the predators in the energy-deficient environments of high altitudes. It is assessed as “vulnerable” since its global population is estimated to be between 2,500–10,000, with its projected decline of at least 10% in 23 years (McCarthy et al., 2017). Similar to other wildlife studies, the snow leopard is also included in Appendix I of CITES (CITES, 2019). Snow leopards are a significant conflict causing wildlife in the High Mountain region of Nepal. They attack the livestock near the livestock sheds in the rangelands of the High Mountain (Karki & Panthi, 2021). Livestock is also the prey of this leopard in the Mountainous region (Oli, Taylor & Rogers, 1993). Due to climate change, its habitat is expected to decline (Aryal et al., 2016). Similarly, about 30% of snow leopard habitat may be lost due to a shifting treeline and consequent shrinking of the alpine zone in the Himalayas (Forrest et al., 2012). Retaliatory killing, poaching, habitat degradation, and prey depletion are considered key factors leading to their population decline (McCarthy et al., 2017).

Species distribution and zonation are essential to identifying ecologically valuable areas for species conservation (Karimi, Brown & Hockings, 2015). Maximum Entropy (MaxEnt) model is a widely used tool for predicting the distribution of the species in Nepal (Aryal et al., 2016; Bista, Panthi & Weiskopf, 2018; Panthi, 2018; Panthi, Aryal & Coogan, 2019; Sharma et al., 2020; Karki & Panthi, 2021; Panthi, Pariyar & Low, 2021). This model demands only presence points (Phillips, Anderson & Schapire, 2006) so this is popular and useful to model the habitat of threatened species, whose occurrence points are not available in large number.

Given the importance and conservation threats of the Asiatic black bear, common leopard, musk deer, and snow leopard in Nepal, it is essential to have comprehensive information on the distribution, habitat, and anthropogenic impacts on these species of interest. Comprehensive information on the distribution and impact of human activities on threatened wildlife is still lacking in the central part of Nepal. Hence, this study attempts to solve these gaps for the Asiatic black bear, common leopard, musk deer, and snow leopard in the Gandaki Province of Nepal using a MaxEnt model. It also assesses suitable habitats of these species in and outside the protected areas. Habitats inside the protected area system face low anthropogenic activities compared to those outside this system. Furthermore, these areas are especially managed for conservation. Therefore we hypothesized that most of the habitat of these four species is covered by the protected area system.

## Methods and materials

### Study area

**Figure 1:**
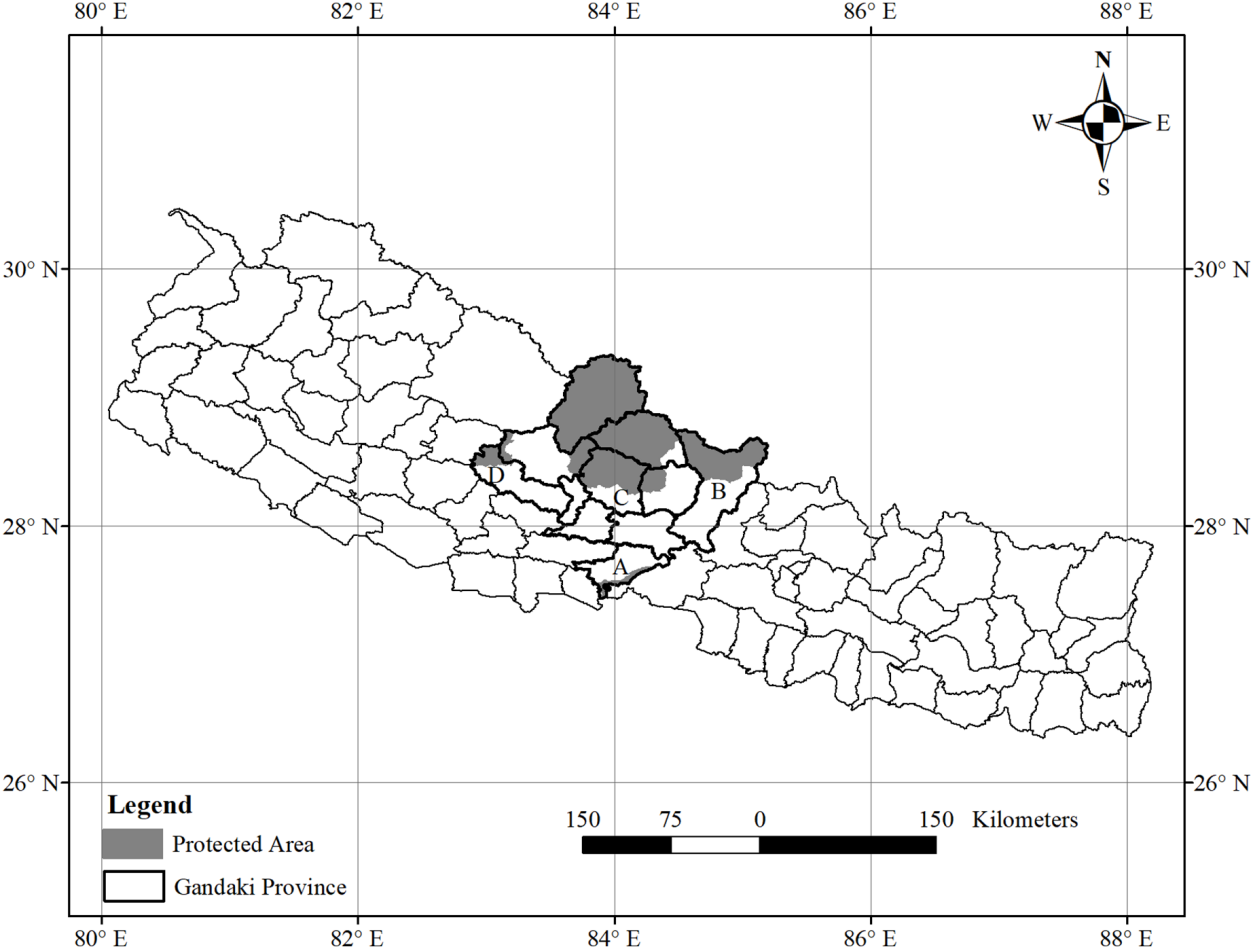
Protected areas covered by study area. (A) Chitwan National Park and Buffer Zone; (B) Manaslu Conservation Area; (C) Annapurna Conservation Area; (D) Dhorpatan Hunting Reserve.

This study was carried out in Gandaki Province, the central part of Nepal (Fig. 1). The province’s total area is 21,976.34 km^2^, which is 14.9% of the total area of Nepal. This province has five distinct geographical regions: Himalaya, high mountains, middle mountains, Siwalik, and Terai. The Himalayan region covers the upper part of the Gandaki Province. Dhawalagiri (8,167 m), Manaslu (8,136 m) and Annapurna (8,091 m) are major mountains of the Gandaki Province. Around 45% of this province is covered by protected areas (Ministry of Industry, Tourism, Forest and Environment of Nepal (MoITFE), 2018). Annapurna Conservation Area, Manaslu Conservation Area, some parts of Dhorpatan Hunting Reserve, and Chitwan National Park are located in this province. ACA Dhorpatan Hunting Reserve and Manaslu Conservation Area are habitats of Himalayan threatened fauna like Asiatic black bear, common leopard, the grey wolf (*Canis lupus*), musk deer, red panda, snow leopard and wild dog (*Cuon alpinus)*. These protected areas are famous for trekking, unique landscape, and mountain biodiversity (Department of National Parks and Wildlife Conservation of Nepal (DNPWC), 2017; Ministry of Industry, Tourism, Forest and Environment of Nepal (MoITFE), 2018). Moreover, the diversity of orchids is high in the Panchase forest conservation area (WWF, 2013). Being situated at the divide of the Eastern and Western floristic regions, the Kali Gandaki Gorge is a recognized corridor for birds to migrate (WWF, 2013).

### Data collection

#### Occurrence points of species

The field survey was conducted from February to June 2020 (approximately 85 days) throughout the Gandaki Province to collect the primary data for the study. The presence of wildlife was recorded based on the direct sight of the particular species or its indirect signs (scats, hairs, and footprints) in the field. Finally, we maintained at least 500 m in each presence point to lessen spatial auto-correlation and retain 62 points for Asiatic black bear, 109 points for common leopard, 62 points for musk deer and 61 points for snow leopard for modelling purposes.

#### Environmental variables

We downloaded environmental variables from online sources. The Digital Elevation Model (DEM) was obtained from the United States Geological Survey (https://earthexplorer.usgs.gov/). This DEM was further used to prepare the slope and aspect with the help of ArcGIS software (release 10.5; Esri, Redlands, CA, USA). Water sources were acquired from the Geofabrik website (https://www.geofabrik.de/data/shapefiles.html) (GEOFABRIK, 2020) and converted to a distance raster file. These data were transformed to ASCII format and spatial resolution of 30 m using ArcGIS (release 10.5; Esri, Redlands, CA, USA) (Table 1).

**Table 1:** Variables used to model the suitable habitat of Asiatic black bear, common leopard, musk deer, and snow leopard.

Source	Category	Variable	Type of variable	Unit
United States Geological Survey	Topographic	Elevation	Continuous	m
		Slope	Continuous	Degree
		Aspect	Continuous	Degree
Geofabrik		Distance to water	Continuous	m
Landsat	Vegetation-related	Annual mean EVI	Continuous	Dimensionless
		Standard deviation of EVI	Continuous	Dimensionless
		Maximum EVI	Continuous	Dimensionless
		Minimum EVI	Continuous	Dimensionless
Global Forest Change		Forest Cover	Continuous	Dimensionless
Geofabrik	Anthropogenic	Distance to settlement	Continuous	m
		Distance to motor road	Continuous	m
		Distance to path	Continuous	m
		Distance to building	Continuous	m
International Centre for Integrated Mountain Development		Land use/land cover	Categorical	m

Herbivores are expected to be directly affected by the vegetation characteristics in the habitat as they form a food source. In contrast, carnivores maybe affected directly (through its effect on shade and hiding areas for predators) or indirectly (through its effect on herbivore prey presence). This study used vegetation-related variables such as forest cover and enhanced vegetation index (EVI). Forest cover data were taken from the global forest change website (Hansen et al., 2013). Also, we used Landsat 8-based EVI time series data for 2018–2019.

This study used anthropogenic datasets, including roads; settlements; and land use land cover (LULC). Vector datasets containing paths and roads were obtained from Geofabrik (https://www.geofabrik.de/data/shapefiles.html) (GEOFABRIK, 2020), and the settlements were obtained from the Department of Survey, Nepal. ArcGIS transformed these files into distance raster files with a distance tool (release 10.5; Esri, Redlands, CA, USA). Distance to paths, roads, and settlements is a distance of a particular pixel of a raster file from paths, roads, and settlements. In addition, LULC data were acquired from the International Centre for Integrated Mountain Development website (ICIMOD; http://www.icimod.org) (Uddin et al., 2015). We selected these environmental variables for modeling based on availability and their importance. The help of existing literatures and expert knowledge identified the importance of variables.

#### Habitat modeling of species

We used Maximum Entropy (MaxEnt) software, version 3.4.1, to predict the species distributions and the anthropogenic impact on these species using species presence points and environmental variables (Table 1) (Phillips, 2017). MaxEnt is a standard and widely used species distribution software. This software is already used to model the suitable habitat of several faunas in Nepal (Aryal et al., 2016; Bista, Panthi & Weiskopf, 2018; Panthi, 2018; KC et al., 2019; Panthi et al., 2019; Sharma et al., 2020). Variance Inflation Factors (VIFs) less than 10 indicate that multicollinearity is not a severe problem (Forthofer, Lee & Hernandez, 2007). Therefore, we calculated the VIFs of variables with the help of R Studio software version 4.1.1 and used those variables having VIF < 10 in the model (Forthofer, Lee & Hernandez, 2007; Panthi, Pariyar & Low, 2021). Of the total presence points, 70% were used for training, and the rest 30% were used for testing and validation. We used 5,000 background points, 10 replications, 1,000 iterations, and subsample replicated run type to model the suitable habitat for all four species. Discriminatory ability showed a slight peak in performance around the default regularization multiplier (Radosavljevic & Anderson, 2014), so we used the default (1) regularization multiplier during the modelling.

#### Accuracy assessment

Accuracy assessment is a prerequisite to understandingand validating the models’ performance. In this study, we used two methods such as threshold-independent and threshold dependent, for model evaluation. The threshold-independent method involves the area under the receiver-operator curve (AUC) elucidating higher than the AUC, better the model performance (Pearce & Ferrier, 2000). AUC values above 0.9 explain excellently, 0.7–0.9 explains moderate, and lower than 0.7 explains poor model performances (Pearce & Ferrier, 2000). However, researchers criticized this method as a classical one, which is supposed to be affected by the non-uniform spatial distribution of samples (Lobo, Jiménez-valverde & Real, 2008). Hence, this study used a threshold-dependent method using the True Skill Statistic (TSS) as a model evaluator (Merow, Smith & Silander, 2013). TSS = Sensitivity + Specificity − 1, and ranges from −1 to 1, where values less than 0 indicate a performance no better than random and 1 indicates a perfect fit of the model (Allouche, Tsoar & Kadmon, 2006; Panthi, Pariyar & Low, 2021). Of the 10 models (0–9 replications) used in the study, TSS was calculated for all models using the PresenceAbsence package in R studio version 4.1.1 and averaged for the final TSS (Jiang et al., 2014; Panthi, Aryal & Coogan, 2019). The sum of sensitivity and specificity was used as a threshold to convert the habitat suitability map (raw output of MaxEnt) into a suitable/unsuitable binary map and to calculate the TSS (Liu, White & Newell, 2013).

## Results

### The suitable habitats of the species

The species-specific suitable habitats and their overlap habitats have been presented in Table 2. In the study area, 6,052 km^2^ was identified as a suitable habitat for the common leopard. Moreover, 5,819 km^2^ was identified as a suitable habitat for Asiatic black bears, 4,447 km^2^ was identified as a suitable habitat for snow leopards, and 1,690 km^2^ was identified as suitable habitat for musk deer. Out of them, more than four-fifth of the suitable habitat of musk deer and three-quarters of the suitable habitat of snow leopards were recorded in protected areas. Most suitable habitats of the common leopards and Asiatic black bears were recorded outside the protected areas, with 88% and 61%, respectively. Substantial suitable habitats for all four species were found in Annapurna Conservation Area. In Annapurna Conservation Area, snow leopards have predicted suitable habitat of 2,472 km^2^, followed by an Asiatic black bear (1,676 km^2^), musk deer (905 km^2^), and common leopard (701 km^2^). While considering habitat overlap between two species, it was observed that overlapped habitat of musk deer and snow leopard inside the protected areas was 85%, followed by Asiatic black bear and musk deer (79%), common leopard and musk deer (78%), Asiatic black bear and snow leopard (63%), common leopard and snow leopard (51%), Asiatic black bear and common leopard (27%). The areas of overlapped habitats for all species were found to be the highest in Annapurna Conservation Area. In addition, the same areas were observed to be suitable for three species of interest simultaneously. These represent overlapped habitat of the Asiatic black bear, musk deer, and snow leopard in Annapurna Conservation Area 355 km^2^; followed by an Asiatic black bear, common leopard, and musk deer (67 km^2^); and common leopard, musk deer and snow leopard (32 km^2^). All four species of Asiatic black bear, common leopard, musk deer, and snow leopard can share an area of 28 km^2^ as their typical suitable habitat.

**Table 2:** Suitable habitat of the species.

S. N.	Habitat of species	Protected area km^2^					Total habitat in PA	Habitat outside the PA	Total habitat	Percentage coverage by PA
		ACA	MCA	DHR	CNP	CNP BZ				
1	Habitat of Asiatic black bear	1,676	393	229			2,298	3,521	5,819	39
2	Habitat of common leopard	701	14	27	0.34	2	745	5,307	6,052	12
3	Habitat of musk deer	905	283	216			1,405	285	1,690	83
4	Habitat of snow leopard	2,472	580	297			3,349	1,098	4,447	75
5	Overlapped habitat of Asiatic black bear and common leopard	536	12	25			572	1,561	2,133	27
6	Overlapped habitat of Asiatic black bear and musk deer	480	162	138			780	206	986	79
7	Overlapped habitat of Asiatic black bear and snow leopard	682	246	78			1,006	599	1,605	63
8	Overlapped habitat of common leopard and musk deer	75	9	11			95	26	122	78
9	Overlapped habitat of common leopard and snow leopard	77	11	4			92	90	182	51
10	Overlapped habitat of musk deer and snow leopard	683	237	112			1,032	179	1,211	85
11	Overlapped habitat of Asiatic black bear, common leopard and musk deer	67	8	10			85	25	111	77
12	Overlapped habitat of Asiatic black bear, musk deer and snow leopard,	355	134	51			541	114	655	83
13	Overlapped habitat of common leopard, musk deer and snow leopard	32	8	1			41	10	51	80
14	Overlapped habitat of Asiatic black bear, common leopard, musk deer and snow leopard	28	7	1			36	10	46	79

**Note:**

ACA, Annapurna Conservation Area; MCA, Manaslu Conservation Area; DHR, Dhorpatan Hunting Reserve; CPN, Chitwan National Park; BZ, Buffer Zone.

### Important variables used in the models

**Figure 2:**
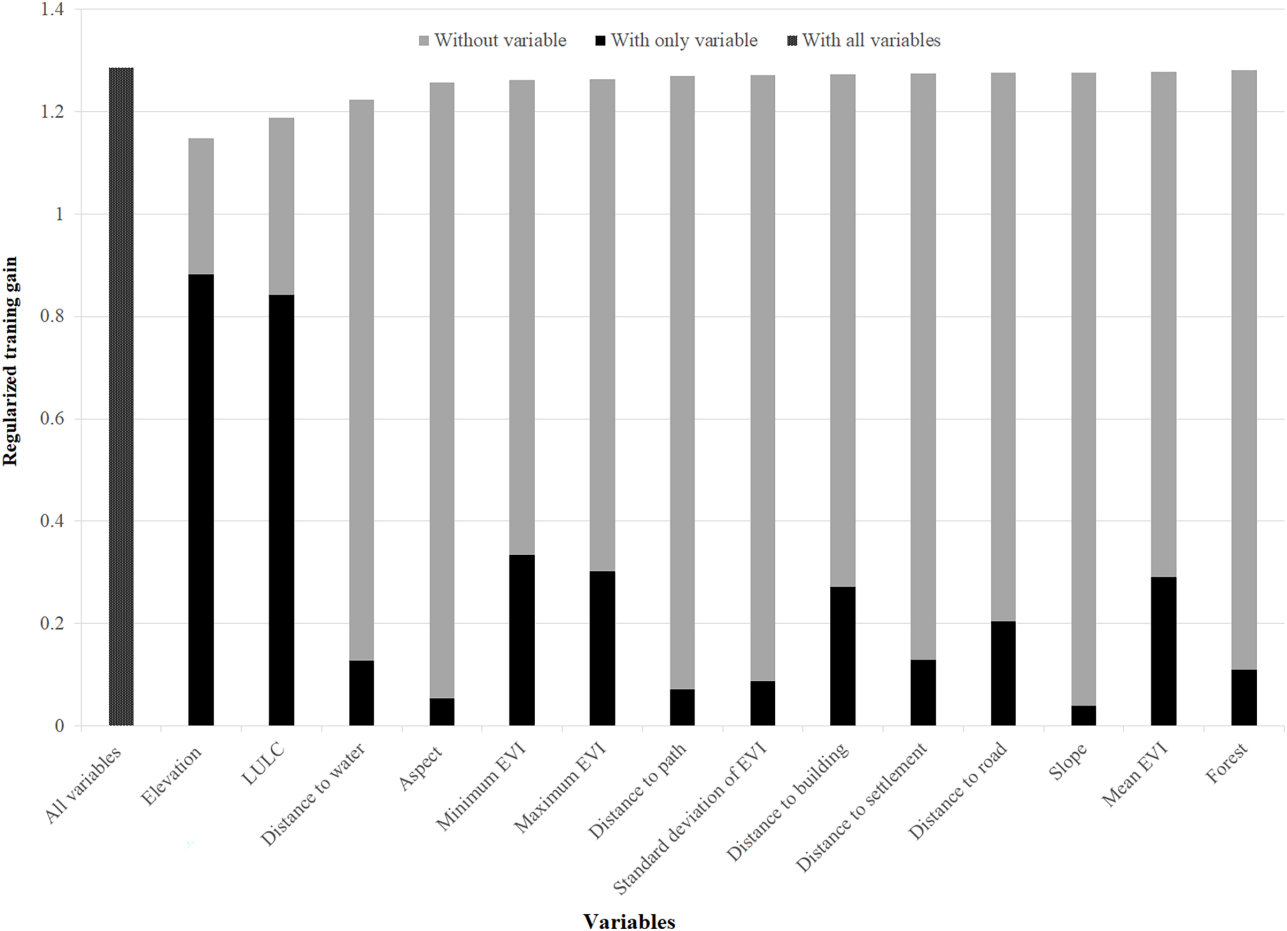
Variables importance for model training for Asiatic black bear. The regularized training gain shows how much better the model distribution fits the presence data relative to a uniform distribution. “Without variable” denotes the effect of removing that single variable from the model “with only variable” denotes the results of the model when an only that variable is run; “with all variables” indicates the results of the model when all variables are run (Phillips, 2017).

Out of 14 variables used in the model, elevation, LULC, distances to water, and minimum EVI were found to be the most important variables determining the habitat suitability of the Asiatic black bear. Anthropogenic variables (distance to path, distance to building, distance to settlement, and distance to road) were moderately essential to model the suitable habitat of this species (Fig. 2).

**Figure 3:**
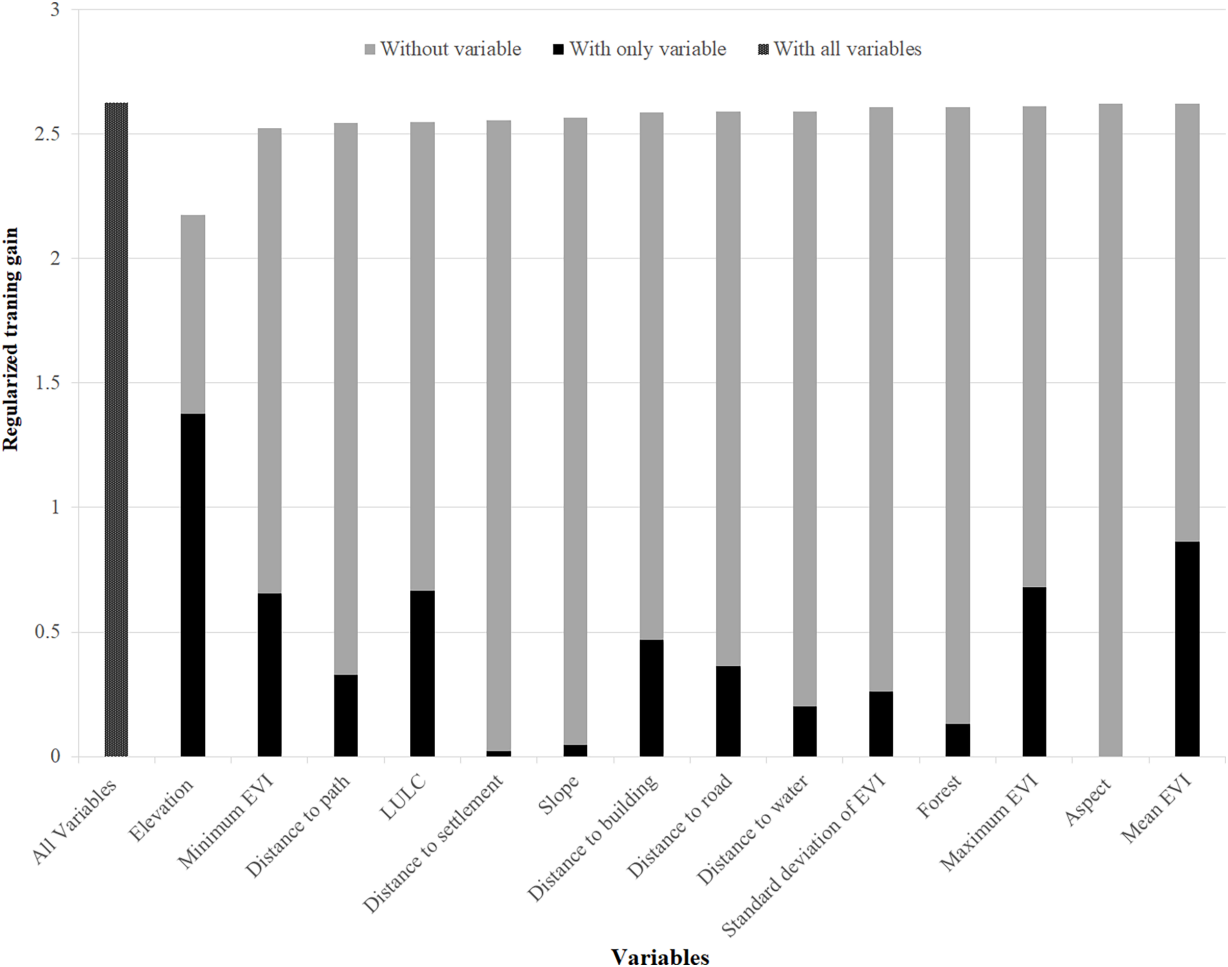
Variables importance for model training for musk deer. The regularized training gain shows how much better the model distribution fits the presence data relative to a uniform distribution. “Without variable” denotes the effect of removing that single variable from the model “with only variable” denotes the results of the model when an only that variable is run; “with all variables” indicates the results of the model when all variables are run (Phillips, 2017).

Similarly, elevation, minimum EVI, distance to a path, and LULC were found to be the most important variables to model the suitable habitat of musk deer (Fig. 3). Moreover, anthropogenic variables (distance to building, distance to settlement, and road distance) were moderately important to model the suitable habitat of this speceis.

**Figure 4:**
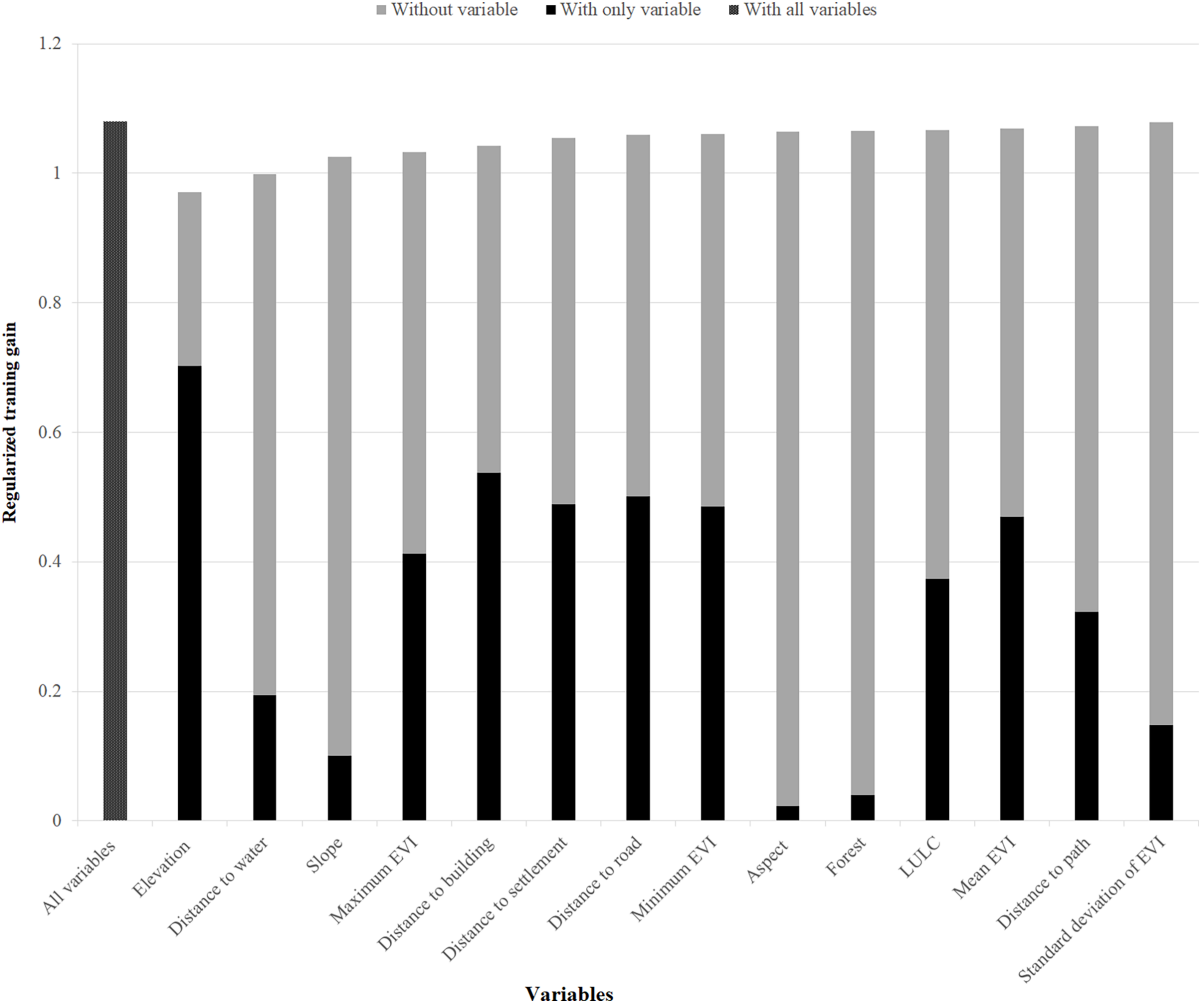
Variables importance for model training for common leopard. The regularized training gain shows how much better the model distribution fits the presence data relative to a uniform distribution. “Without variable” denotes the effect of removing that single variable from the model “with only variable” denotes the results of the model when an only that variable is run; “with all variables” indicates the results of the model when all variables are run (Phillips, 2017).

Also, elevation, distances to water, slope, and maximum EVI were found to be the most important variables to model the suitable habitat of common leopard (Fig. 4). Furthermore, anthropogenic variables (distance to building, distance to settlement, distance to road, and distance to path) were moderately important to model the suitable habitat of this speceis.

**Figure 5:**
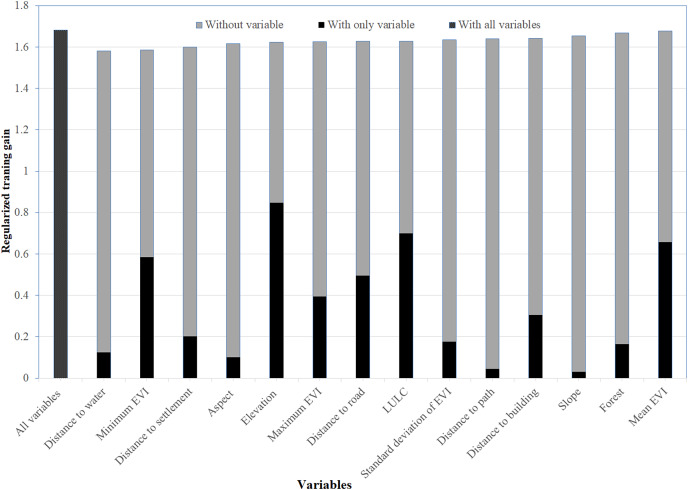
Variables importance for model training for snow leopard. The regularized training gain shows how much better the model distribution fits the presence data relative to a uniform distribution. “Without variable” denotes the effect of removing that single variable from the model “with only variable” denotes the results of the model when an only that variable is run; “with all variables” indicates the results of the model when all variables are run (Phillips, 2017).

Of the 14 variables used in the model, distance to water, minimum EVI, distances to settlement, and aspect were found to be the most important variables to model the suitable habitat of snow leopard (Fig. 5). Anthropogenic variables such as distance to road, distance to path, distance to building, and LULC were moderately important to model the suitable habitat of this speceis.

### Response of habitat suitability of species to anthropogenic variables

**Figure 6:**
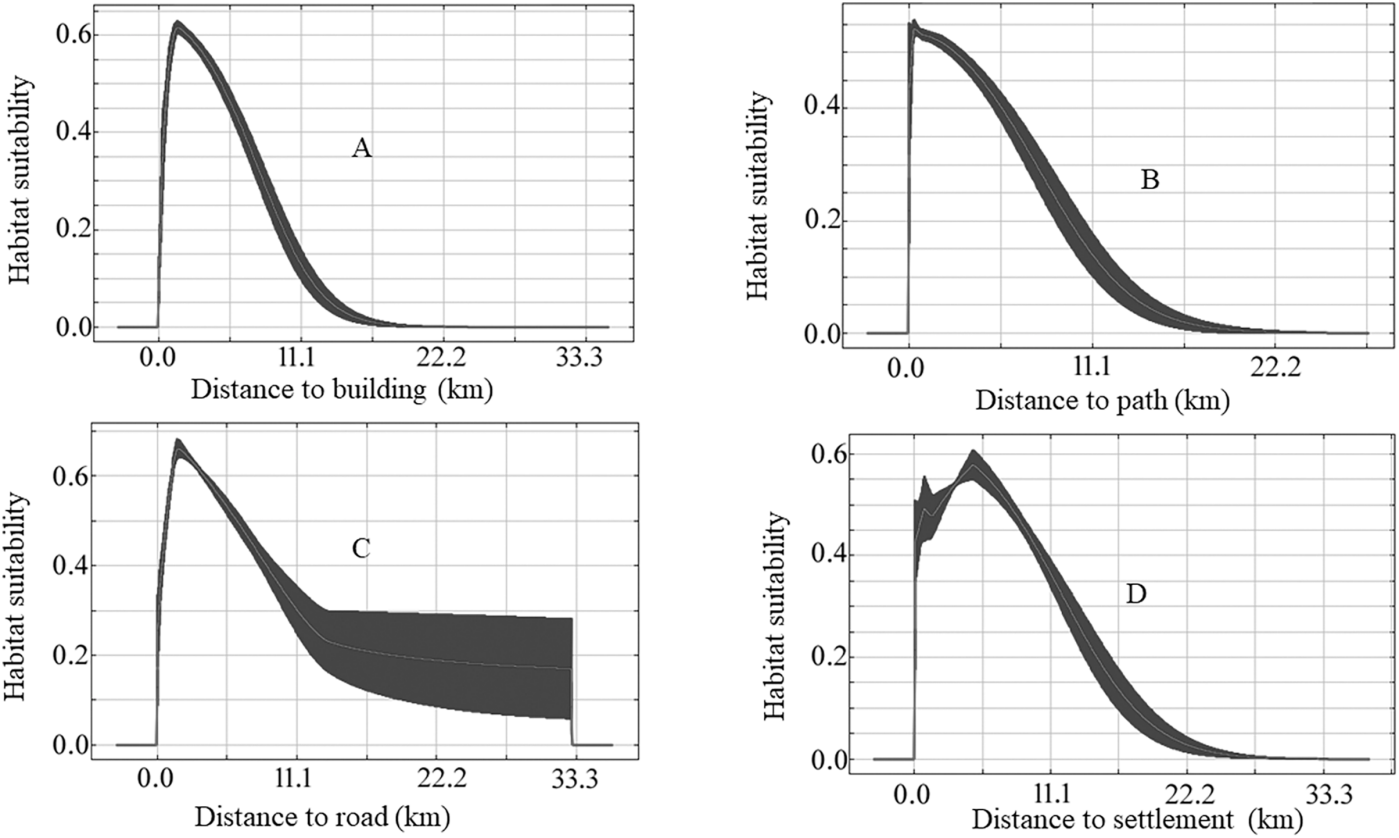
Response of habitat suitability of Asiatic black bear to anthropogenic variables. Darker areas of figures are variations of result during the different run. (A) Response of habitat suitability of Asiatic black bear to distance to buildings; (B) response of habitat suitability of Asiatic black bear to distance to path; (C) response of habitat suitability of Asiatic black bear to distance to road; (D) response of habitat suitability of Asiatic black bear to distance to settlement.

**Figure 7:**
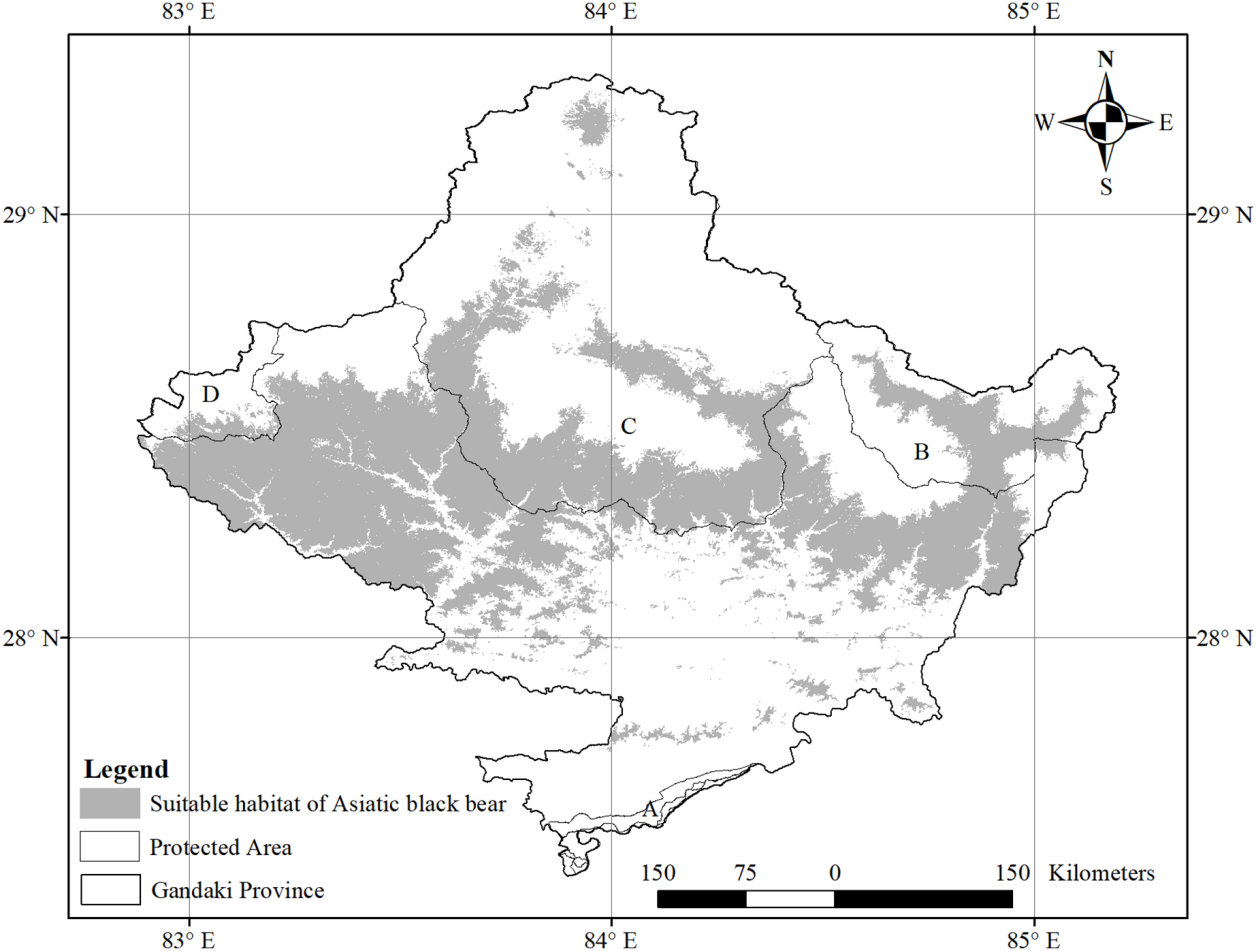
Suitable habitat of Asiatic black bear. (A) Chitwan National Park and its Buffer Zone; (B) Manaslu Conservation Area; (C) Annapurna Conseravtion Area; (D) Dhorpatan Hunting Reserve.

**Figure 8:**
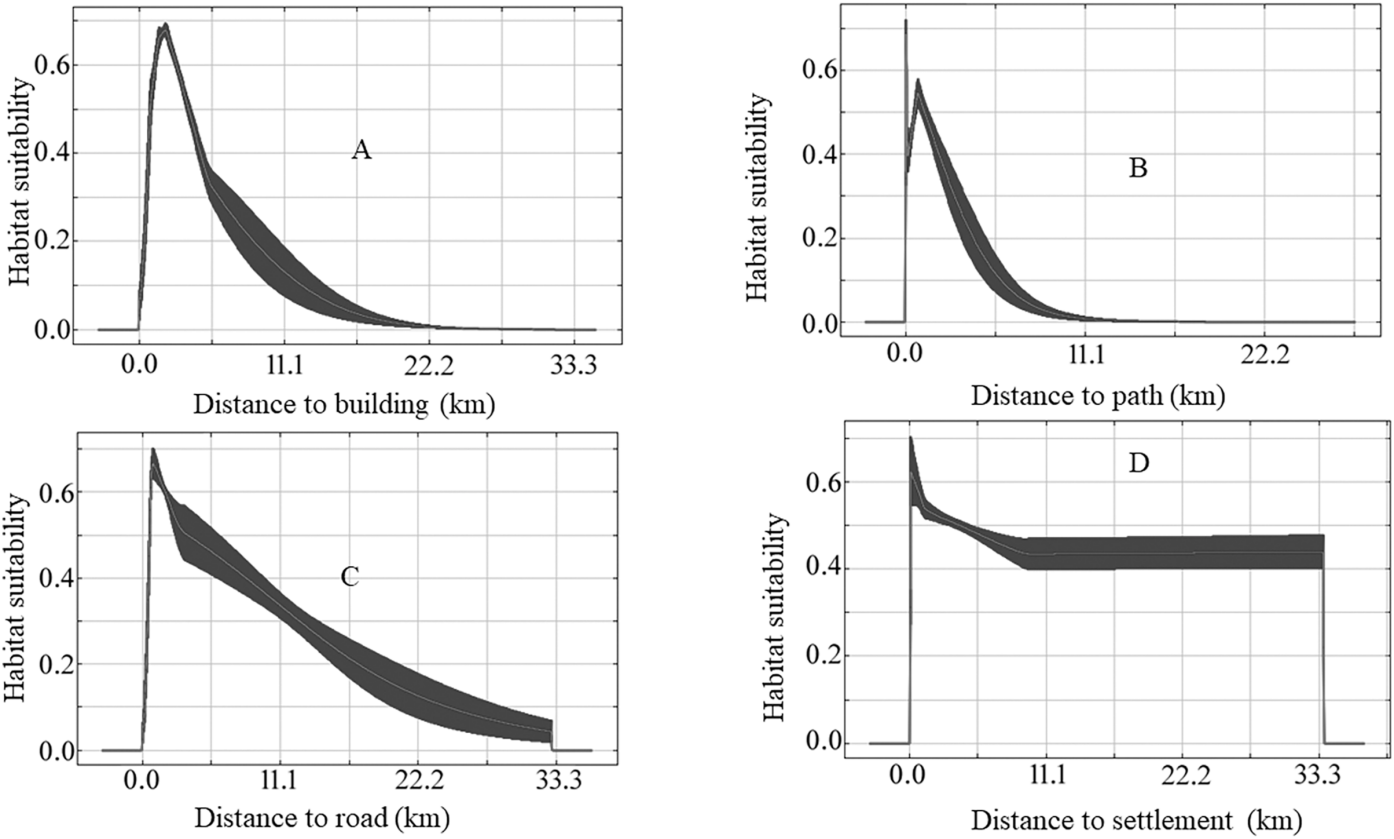
Response of habitat suitability of musk deer to anthropogenic variables. Darker areas of figures are variations of result during the different run. (A) Response of habitat suitability of musk deer to distance to buildings; (B) response of habitat suitability of musk deer to distance to path; (C) response of habitat suitability of musk deer to distance to road; (D) response of habitat suitability of musk deer to distance to settlement.

**Figure 9:**
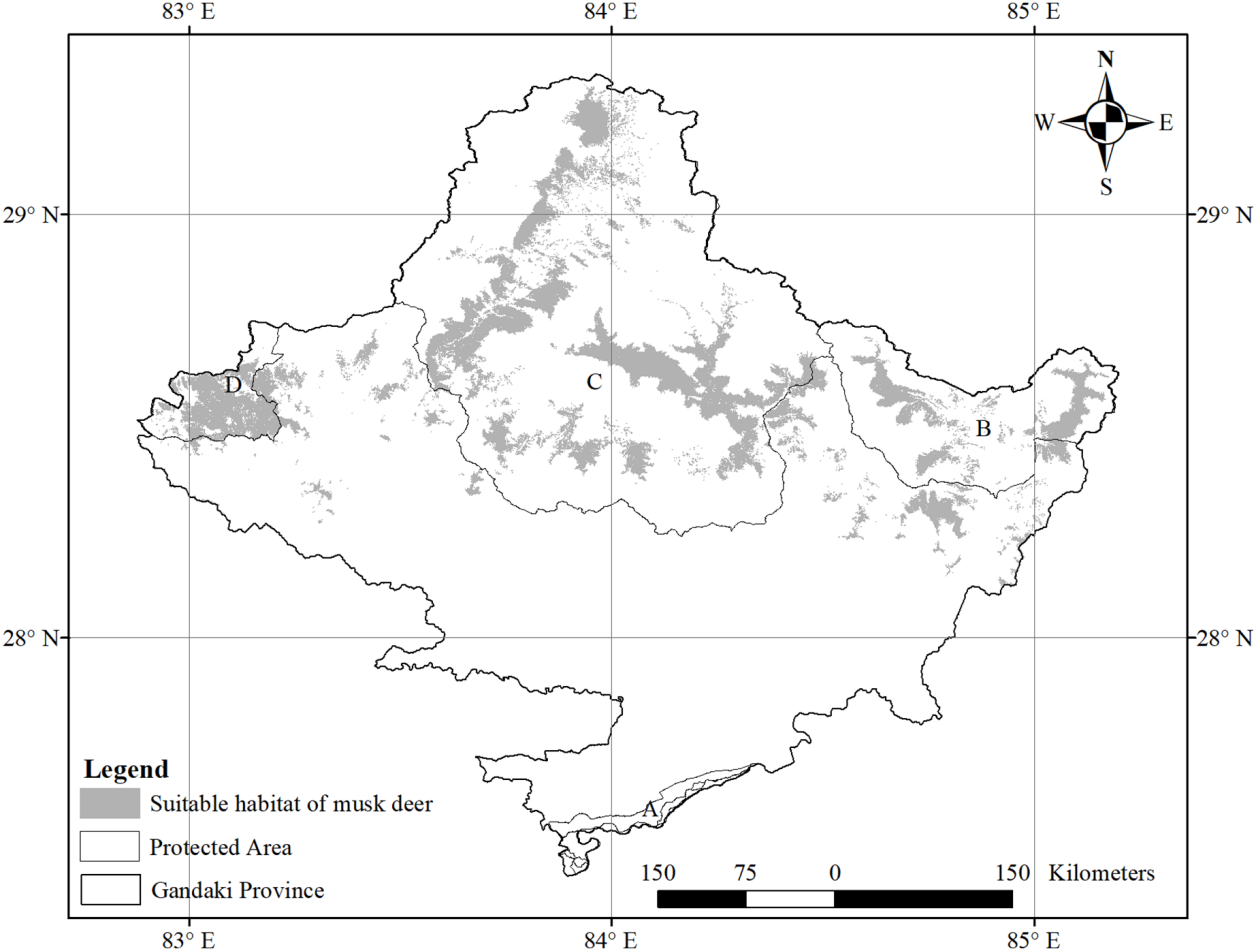
Suitable habitat of musk deer. (A) Chitwan National Park and its Buffer Zone; (B) Manaslu Conservation Area; (C) Annapurna Conseravtion Area; (D) Dhorpatan Hunting Reserve.

**Figure 10:**
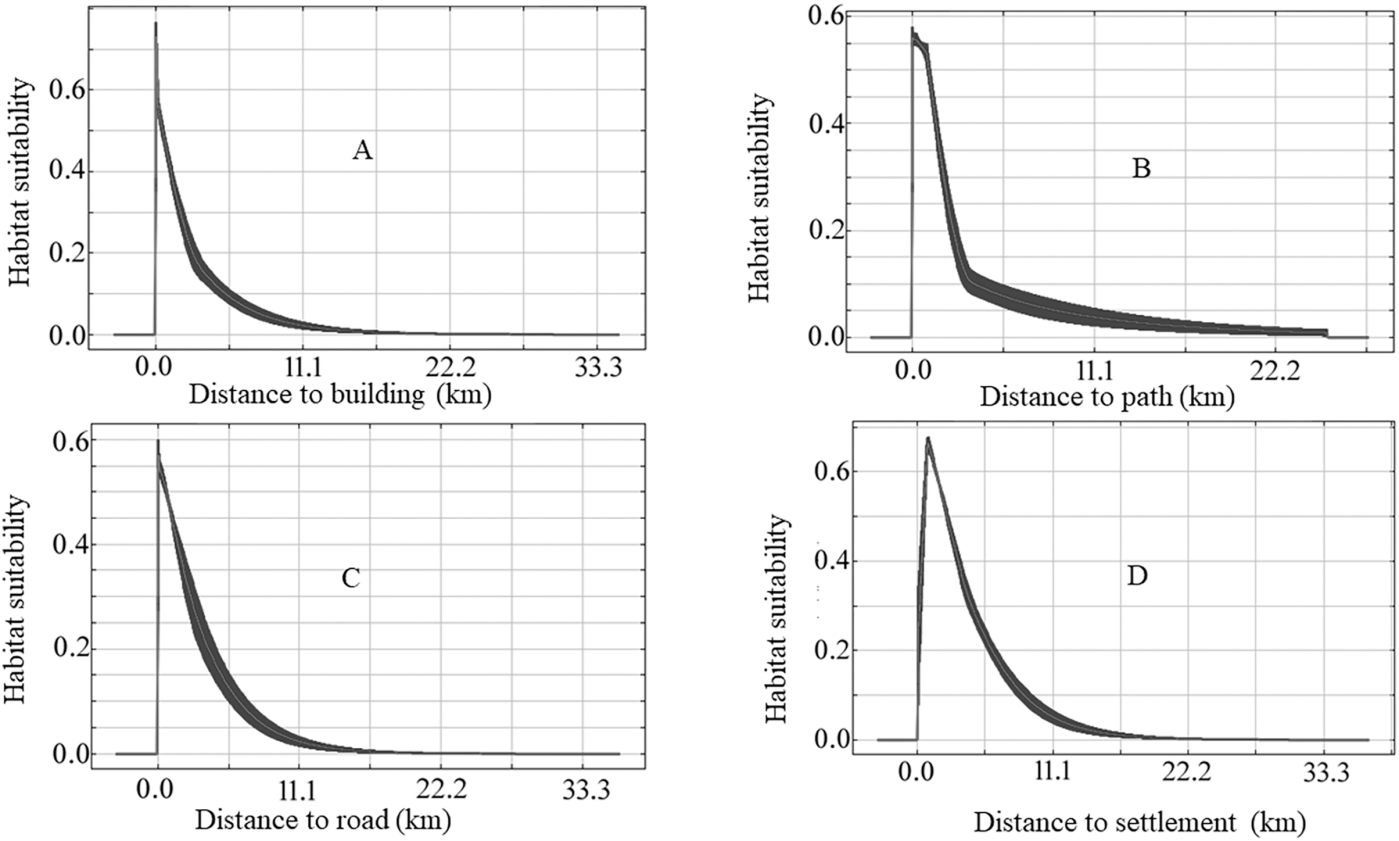
Response of habitat suitability of common leopard to anthropogenic variables. Darker areas of figures are variations of result during the different run. (A) Response of habitat suitability of common leopard to distance to buildings; (B) response of habitat suitability of common leopard to distance to path; (C) response of habitat suitability of common leopard to distance to road; (D) response of habitat suitability of common leopard to distance to settlement.

**Figure 11:**
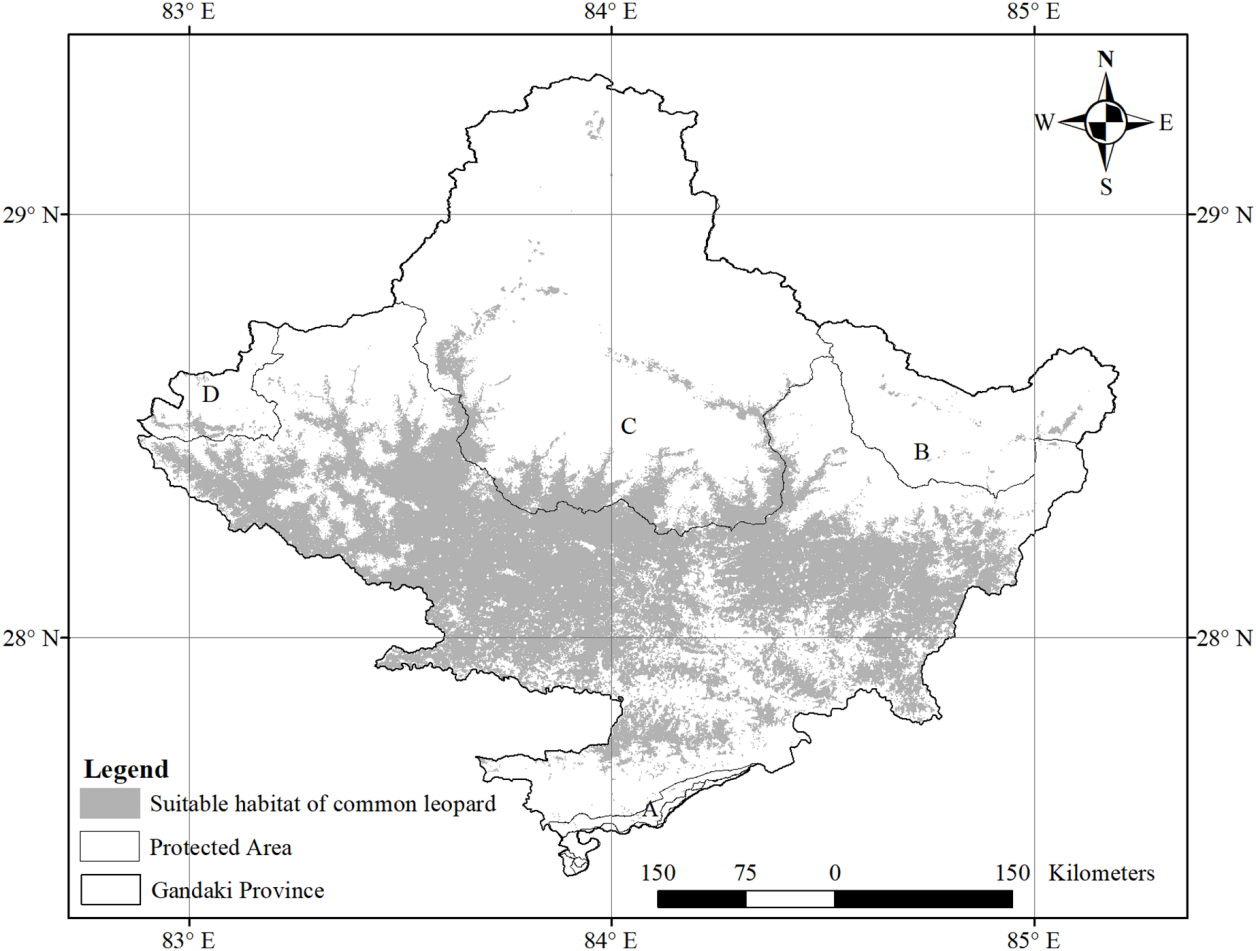
Suitable habitat of common leopard. (A) Chitwan National Park and its Buffer Zone; (B) Manaslu Conservation Area; (C) Annapurna Conseravtion Area; (D) Dhorpatan Hunting Reserve.

**Figure 12:**
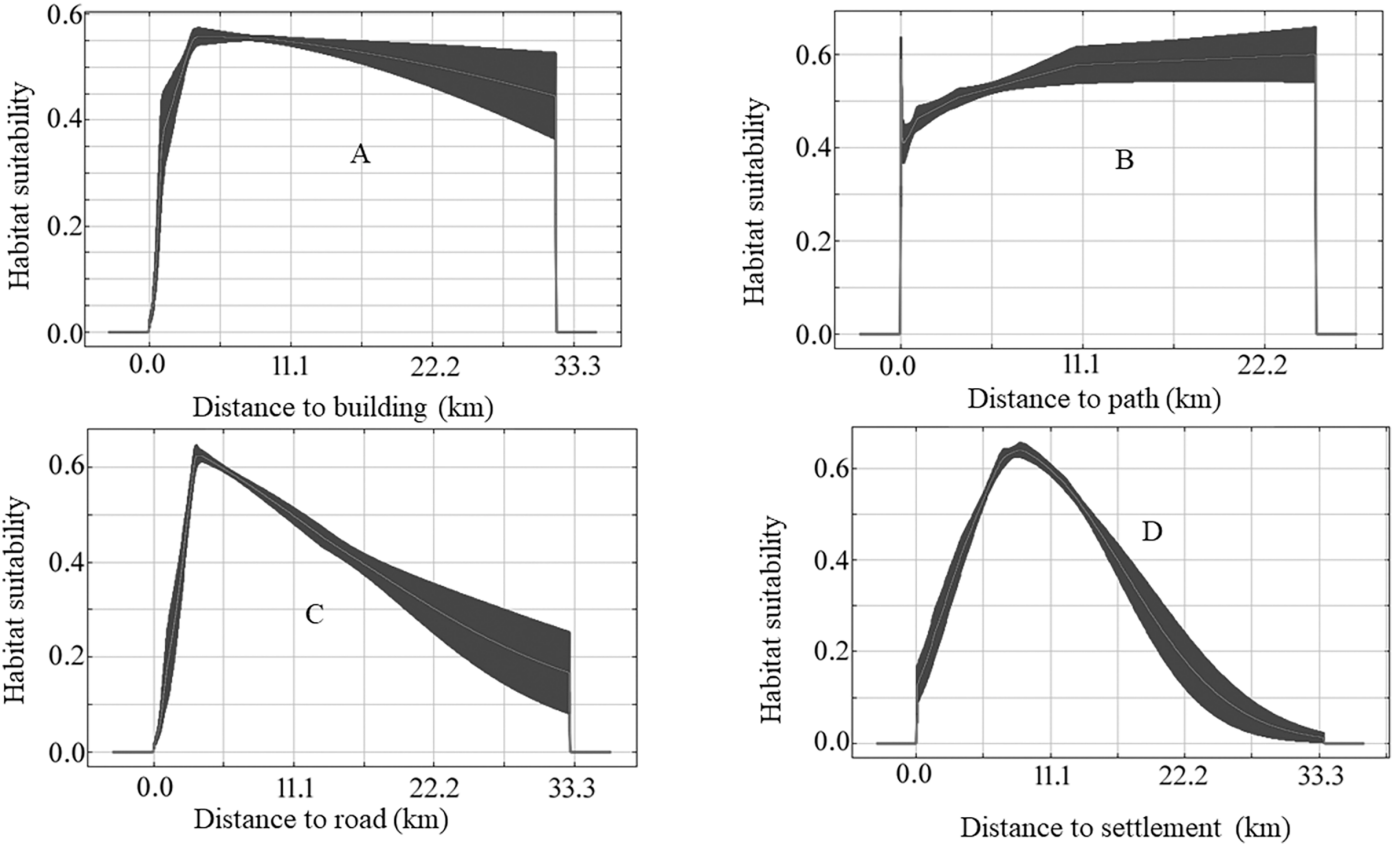
Response of habitat suitability of snow leopard to anthropogenic variables. Darker areas of figures are variations of result during the different run. (A) Response of habitat suitability of snow leopard to distance to buildings; (B) response of habitat suitability of snow leopard to distance to path; (C) response of habitat suitability of snow leopard to distance to road; (D) response of habitat suitability of snow leopard to distance to settlement.

**Figure 13:**
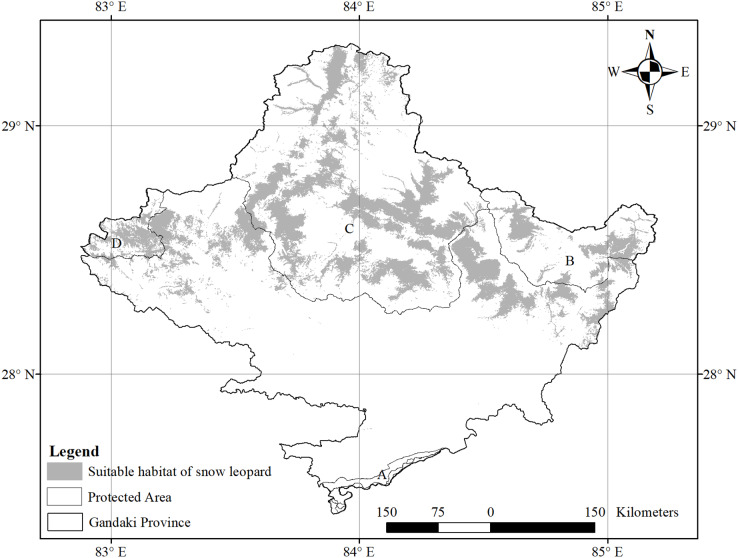
Suitable habitat of snow leopard. (A) Chitwan National Park and its Buffer Zone; (B) Manaslu Conservation Area; (C) Annapurna Conseravtion Area; (D) Dhorpatan Hunting Reserve.

The species-specific models of Asiatic black bears indicate that their existing habitats are near four anthropogenic variables such as building, path, road, and settlement. The habitat suitability of this species increases with decreasing distance from buildings, paths, roads, and settlements (Fig. 6). The map of suitable habitats of this species is shown (Fig. 7). The species-specific models of musk deers indicate that their existing habitats are near four anthropogenic variables such as building, path, road, and settlement, and habitat suitability of this species increases with decreasing distance from building, path, road, and settlement (Fig. 8). The map of suitable habitats of this species is shown (Fig. 9). The species-specific models of common leopards indicate that their existing habitats are near four anthropogenic variables such as building, path, road, and settlement, and habitat suitability of this species increases with decreasing distance from building, path, road, and settlement (Fig. 10). The map of suitable habitats of this species is shown (Fig. 11). In the case of snow leopards, habitat suitability is the maximum at a certain distance from the building, road, and settlements (Fig. 12). The map of suitable habitats of this species is shown (Fig. 13).

### Model accuracy

The accuracies of the models were found to be relatively suitable for all models. We obtained 0.828 ± 0.053 AUC and 0.607 ± 0.086 TSS for the model, which predicts the suitable habitat of the Asiatic black bear (Table 3). Similarly, we obtained 0.964 ± 0.016 AUC and 0.864 ± 0.072 TSS for the model predicting the suitable habitat of musk deer (Table 4); 0.858 ± 0.040 AUC and 0.608 ± 0.081 TSS for the model predicting suitable habitat of common leopard (Table 5); and 0.879 ± 0.014 AUC and 0.655 ± 0.038 TSS for the model predicting suitable habitat of snow leopard (Table 6).

**Table 3:** Thresholds and accuracies of different replications during the modelling of habitat suitability of Asiatic black bear in 10 (0–9) replications.

S. N.	Accuracy	0	1	2	3	4	5	6	7	8	9	Average	Std
1	Threshold	0.090	0.260	0.210	0.260	0.140	0.240	0.160	0.070	0.150	0.190	0.177	0.067
2	TSS	0.500	0.723	0.643	0.621	0.659	0.716	0.612	0.460	0.584	0.556	0.607	0.086
3	AUC	0.798	0.880	0.853	0.852	0.860	0.886	0.830	0.705	0.810	0.809	0.828	0.053

**Table 4:** Thresholds and accuracies of different replications during the modelling of habitat suitability of musk deer in 10 (0–9) replications.

S. N.	Accuracy	0	1	2	3	4	5	6	7	8	9	Average	Std
1	Threshold	0.040	0.260	0.110	0.290	0.110	0.120	0.120	0.010	0.060	0.040	0.116	0.093
2	TSS	0.815	0.956	0.917	0.804	0.922	0.928	0.831	0.724	0.894	0.849	0.864	0.072
3	AUC	0.959	0.985	0.978	0.948	0.972	0.975	0.954	0.933	0.968	0.969	0.964	0.016

**Table 5:** Thresholds and accuracies of different replications during the modelling of habitat suitability of common leopard in 10 (0–9) replications.

S. N.	Accuracy	0	1	2	3	4	5	6	7	8	9	Average	Std
1	Threshold	0.180	0.260	0.130	0.220	0.080	0.170	0.130	0.370	0.460	0.430	0.243	0.134
2	TSS	0.595	0.617	0.593	0.540	0.486	0.577	0.539	0.726	0.736	0.667	0.608	0.081
3	AUC	0.847	0.867	0.845	0.826	0.800	0.838	0.829	0.917	0.920	0.887	0.858	0.040

**Table 6:** Thresholds and accuracies of different replications during the modelling of habitat suitability of snow leopard in 10 (0–9) replications.

S. N.	Accuracy	0	1	2	3	4	5	6	7	8	9	Average	Std
1	Threshold	0.180	0.090	0.130	0.100	0.090	0.050	0.160	0.130	0.400	0.190	0.152	0.098
2	TSS	0.603	0.624	0.675	0.688	0.680	0.601	0.661	0.637	0.658	0.720	0.655	0.038
3	AUC	0.870	0.879	0.877	0.891	0.886	0.855	0.881	0.865	0.885	0.903	0.879	0.014

## Discussions

This comprehensive study identifies the suitable habitat of the four threatened Himalayan wildlife in Gandaki Province, Nepal, with the help of MaxEnt modelling. The total suitable habitat of the Asiatic black bear is found to be 5,819 km^2^. Similarly, suitable habitats for common leopards, musk deer, and snow leopards are 6,052, 1,690, and 4,447 km^2^, respectively. Unlike our hypothesis, many patches of suitable habitat for the threatened fauna are identified outside the protected areas.

Our study shows that most of the suitable habitat of Asiatic black bears is found outside the protected area (61%). Similarly, Mohammadi et al. (2021) identified most brown bears’ habitat outside the protected area in Iran. It was reported in the village that the black bears mainly inhabit the forests and visit agricultural fields close to the forests (Ali et al., 2017). The Asiatic black bear shares a habitat with the rest of the studied species in the study area. Previous studies also recorded the habitat sharing of Asiatic black bears with red pandas in Makalu Barun National Park, Nepal (Bista, Panthi & Weiskopf, 2018).

Most of the signs of Asiatic black bears have been recorded at an elevation ranging from 1,900 to 3,100 m (Bista & Aryal, 2013). Elevation and bioclimatic variables were major contributors to model the habitat of the Asiatic black bear in the Gandaki River Basin. A similar study area of this study used bioclimatic variables to predict the climate change effect (Rai et al., 2022). However, our study did not use these bioclimatic variables considering a coarse resolution to model the wildlife habitat in a small study area. So we might find the elevation as the most important variable, which could work as a proxy for climatic variables. Water resource availability was the most important variable in modeling the brown bear distribution in Iran (Ansari & Ghoddousi, 2018).

In recent years, anthropogenic activities and infrastructure development, such as road construction, have jeopardized the habitats and life forms of Asian elephants (Sharma et al., 2020). Similarly, the habitat of Asiatic black bears is declining due to human encroachment (Escobar, Awan & Qiao, 2015). Cereal crops such as maize and millet were identified in the diet of Asiatic black bears in two protected areas of Nepal (Panthi, Aryal & Coogan, 2019). This evidence proves that the Asiatic black bear is facing anthropogenic impact.

We found elevation, distances to water, slope, and maximum EVI were found to be the most important variables in modeling the suitable habitat for common leopard. Leopards showed a high tolerance to infrastructure and fragmented patches of forests used by humans for agriculture and other land uses (Sarkar et al., 2018). We also find anthropogenic variables (distance to building, distance to settlement, distance to road, and distance to path) moderately important to model the suitable habitat of common leopards. The common leopards are widely distributed wild cats and occupy various habitats which includes rainforest, deserts, fringes of urban areas and remote mountains (Dickman & Marker, 2005; Baral et al., 2021). They are known to exhibit a high tolerance to human activities in their habitat (Athreya et al., 2013). In Nepal, many common leopards were recorded in community-managed forests outside protected areas, threatening human lives living closer to the forests (Baral et al., 2021). Habitats of the common leopard and tiger (*P. tigris*) overlapped in India (Rather, Kumar & Khan, 2020). The leopard prefers agricultural land until it is covered with bush and forest, as these areas have a greater affinity towards the prey base (Maharjan et al., 2017). Among the six variables chosen deliberately for the model, the most important variables were settlement area, sparse forest, bush, and roads (Maharjan et al., 2017). Habitat suitability modeling results of the common leopard in a representative Himalayan landscape of Kailash Sacred Landscape of India assured that the common leopard habitat is not only influenced by topographic and environmental features but also by their combined spatial arrangements at different spatial scales (Sarkar et al., 2018).

Avoidance of humans was the primary influence on the distribution of the Persian leopard (*Panthera pardus saxicolor*) in the Montane areas of West Asia (Farhadinia et al., 2015). The contribution of the variable “distance from settlement area” was the highest (52.4%) to impact the model while calculating the habitat suitability of the common leopard in Shivapuri Nagarjun National Park (Maharjan, 2013). The probability of the presence of *P. pardus* also increases up to a distance of 750 m and whereas decreases up to 1,200 m (Maharjan et al., 2017). Also, anthropogenic factors such as the extent of 31 spots of deforestation (decrease in forest cover) negatively affected leopard occupancy in Nepal’s Terai Arc Landscape region (Thapa et al., 2020).

We identified most of the habitat of musk deer inside the protected areas. In Nepal, very few studies were conducted to identify the habitat of musk deer. Based on the government documents, Annapurna Conservation Area, Manaslu Conservation Area, and Dhorpatan Hunting Reserve are the habitat of musk deer (Department of National Parks and Wildlife Conservation of Nepal (DNPWC), 2017). Musk deer prefers high-altitude regions above 2,500 m and is found mostly in the central and eastern parts of the country (Lamsal et al., 2018). In our study, most of the habitat of the musk deer falls inside the Annapurna Conservation Area, Manaslu Conservation Area, and Dhorpatan Hunting Reserve. Most parts of these protected areas are situated above the 2,500 m elevation. Temperature is the most influential variable in the distribution of musk deer in Nepal Himalaya since annual mean temperature and isothermality are two key contributors to the model (Lamsal et al., 2018). The vegetation type/land use was the most important variable for the habitat suitability assessment of the Himalayan musk deer in Kedarnath Wildlife Sanctuary (Nandy, Lakshmi & Kushwaha, 2020). Rocky areas, ridges, mixed coniferous forest (Red pine and Mongolian oak), southern and southeastern slopes, elevation above 800 m, and distance from water course less than 300 m were dominant habitat types of Siberian musk deer. This study also found DEM and mean EVI to be the most important variables determining habitat suitability of musk deer. In this study, temperature was not taken as the separate variable but accompanied by elevation. Musk deer population has been declined in its native regions owing to various anthropogenic threats such as habitat fragmentation and illegal hunting (Khanal, 2020). For the selection of habitat of the musk deer habitat types, fuel wood and timber cutting, rock cover, litter cover and distance to settlements were the main factors (Shrestha & Xiuxiang, 2014). Habitat of the musk deer was associated with contrasting species composition of trees and forbs, and certain species of shrubs. Main causes for reducing the population of musk deer was poaching and human induced habitat alterations (Shrestha & Xiuxiang, 2014).

Our study claims that most of the suitable habitat of the snow leopard was recorded inside the protected area. Based on the habitat model, Jackson & Ahlborn (1989) concluded that 65% of Nepal’s snow leopard population is inhabited outside the protected areas. After that study, two conservation areas (Annapurna and Manaslu) were established in our study area. Now, most of the snow leopard habitat is covered by these conservation areas. Annapurna and Manaslu Conservation areas are snow leopard habitats (Aryal et al., 2014; Department of National Parks and Wildlife Conservation of Nepal (DNPWC), 2017). DEM and LULC were found to be the most important variables determining the habitat suitability of snow leopards. The snow leopard’s presence ranged from 2,965 to 5,831 m and a maximum distance to water of 1,575 m (Watts, McCarthy & Namgail, 2019). However, in our study, elevation is not identified as the most important environmental variable (Fig. 5). Land cover and aspect contributed less to the model, and land cover and prey were the least important ones (Watts, McCarthy & Namgail, 2019). In our study, distance to water, minimum EVI, distances to settlement, and aspect are found to be the most important variables in modeling the suitable habitat of snow leopards.

Similarly, we observed the habitat overlap of snow leopards with the other three studied species in the study area. Wild herbivores, marmots, and livestock (blue sheep and other wild ungulates) were major snow leopard prey (Oli, Taylor & Rogers, 1993). Our study identified 1,211 km^2^ as overlapped snow leopard and musk deer habitats. In overlapped habitats, musk deer may be prey for the snow leopard. Snow leopards are significant conflict-causing wildlife in the High Mountain region of Nepal; they attack the livestock near the livestock sheds in rangelands of the High Mountain region (Oli, Taylor & Rogers, 1993; Karki & Panthi, 2021). Retaliatory killing, poaching, smuggling, habitat degradation, and prey depletion are considered key factors leading to their population decline (McCarthy et al., 2017).

We found the anthropogenic impact on the habitat of our studied animals in the study area. The habitat suitability of Asiatic black bears, common leopards, and musk deer increases with decreasing distance from buildings, paths, roads, and settlements (Figs. 6, 8 and 10). This means these animals’ habitats are near the path, road buildings, and settlements. Therefore we can assume that these three wildlife face an enormous anthropogenic impact on their habitat. In the case of snow leopards, habitat suitability is maximum at a certain distance from the building, road, and settlements (Fig. 12). This information can indicate that the snow leopard is facing a moderate anthropogenic impact on its habitat. Habitats of snow leopards are grassland and bare land of the High Himalayan region, which are very far from human activities. Therefore anthropogenic variables might be less influential in predicting this species’ habitat.

## Conclusion

Some species can utilize a wider range of habitats than others, resulting in a more extensive suitable habitat. Unlike our hypothesis, most of the areas of suitable habitat for common leopards and Asiatic black bears were outside the protected areas. Suitable habitats of musk deer and snow leopards were inside the protected areas. This spatial distribution of the suitable habitat of the threatened fauna suggests managing the wildlife habitat outside the protected areas. Annapurna Conservation Area, Dhorpatan Hunting Reserve, and Manaslu Conservation Area are situated in the mountainous region of Gandaki Province. These protected areas cover most of the suitable musk deer and snow leopard habitats. Habitats located inside and outside the protected areas are equally important for this wildlife. Therefore, we recommend managing these animals’ habitats inside and outside protected areas. This study emphasizes maintaining land use land cover based on the need of particular animal species, which is crucial to halt the rate of population decrease and an important step to biodiversity conservation. Furthermore, protecting and managing water sources is also recommended to conserve the wildlife in the study area.

## Supplemental Information

10.7717/peerj.16085/supp-1 Supplemental Information 1 Photos of scat, pugmark and hair.

10.7717/peerj.16085/supp-2 Supplemental Information 2 Dataset.

## Additional Information and Declarations

### Data Availability

The following information was supplied regarding data availability: The coordinates taken for each species are available in the Supplemental File.
