# Supplementary figures and images for: Habitat suitability of four threatened Himalayan species: Asiatic black bear, common leopard, musk deer, and snow leopard
Source: PeerJ. 2023 Sep 25;11:e16085. doi: 10.7717/peerj.16085 (PMC10538300; doi:10.7717/peerj.16085)

Hair of Musk Deer


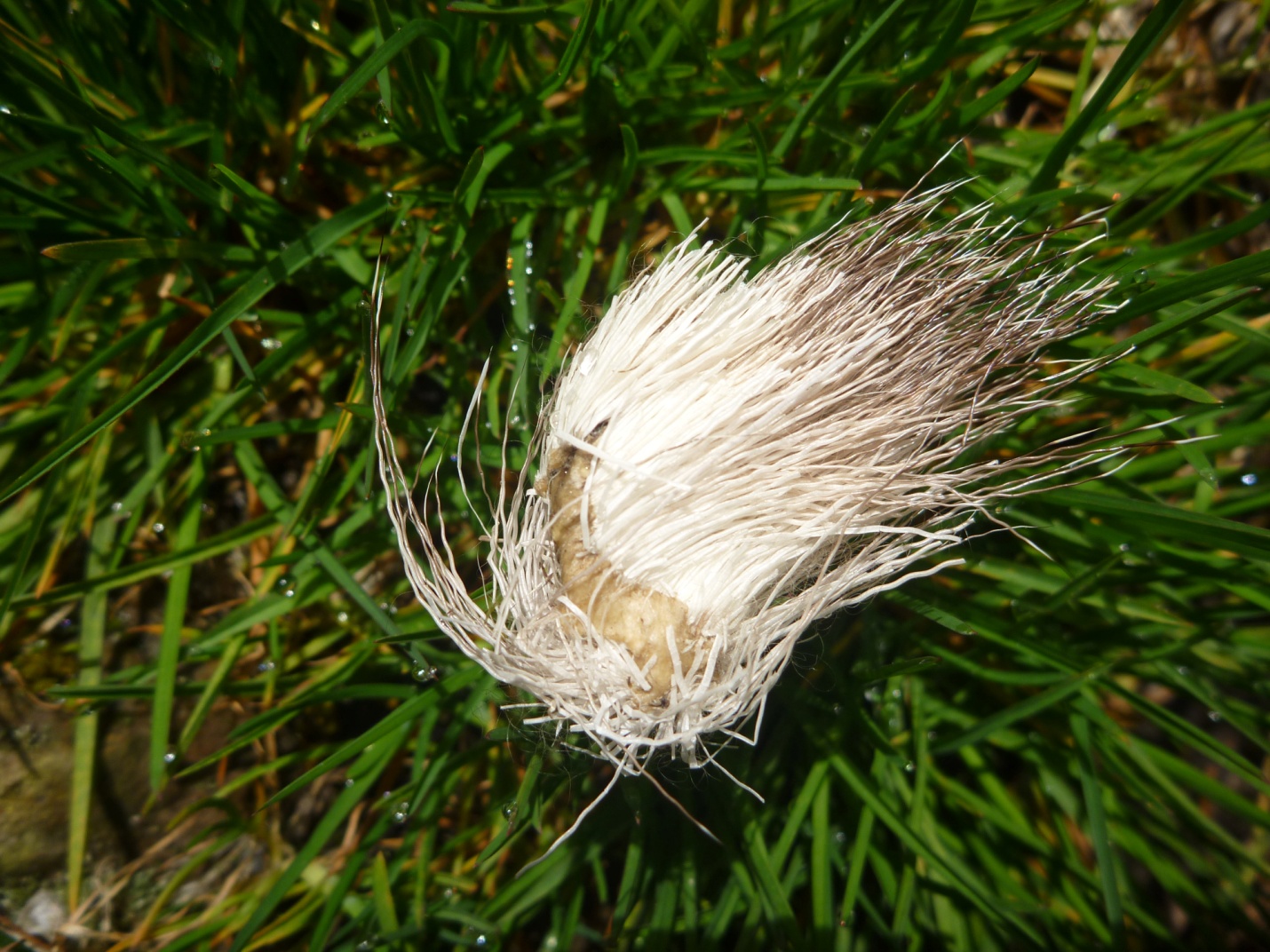


Pug Mark of Asiatic Black Beer


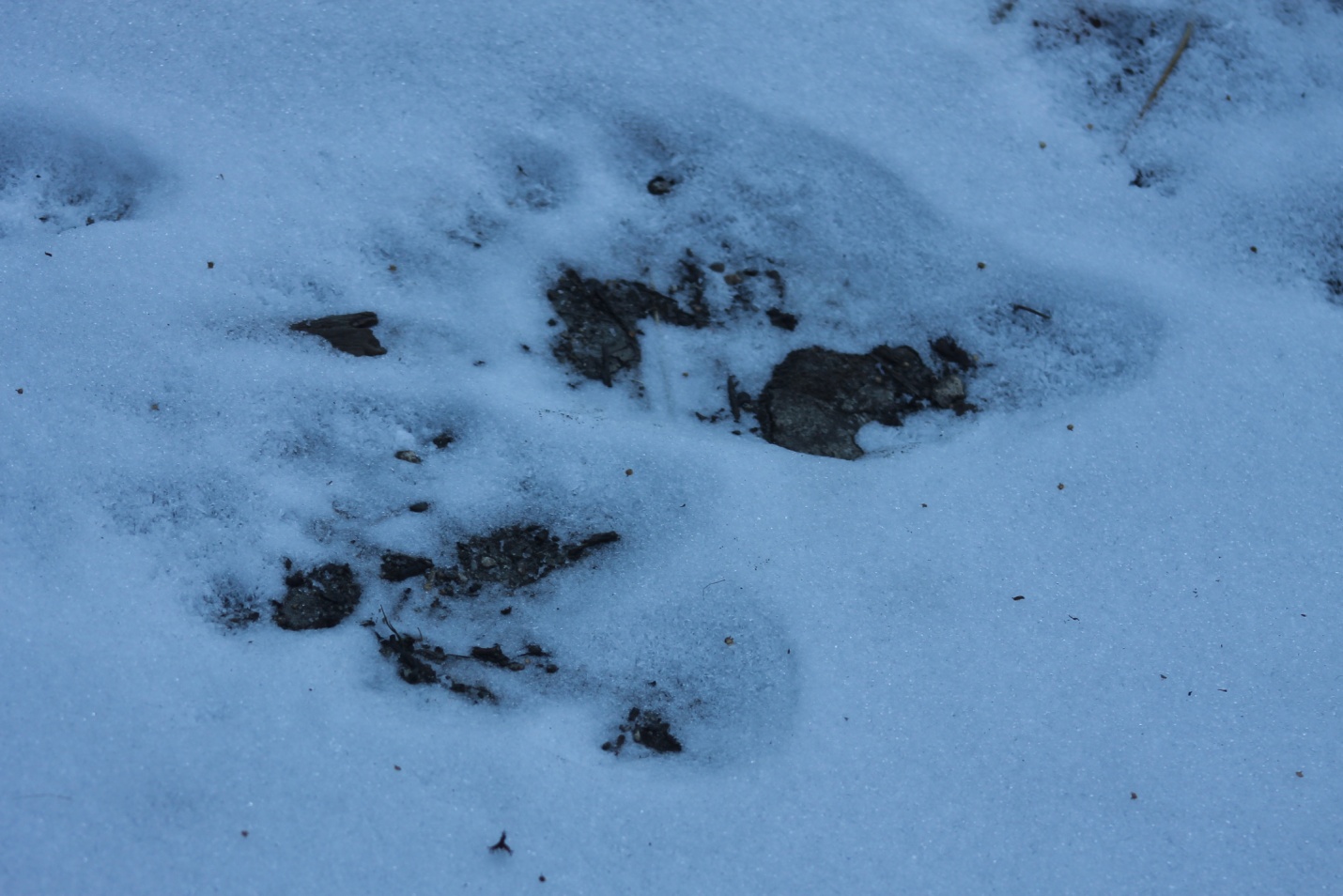


Scat of snow leopard


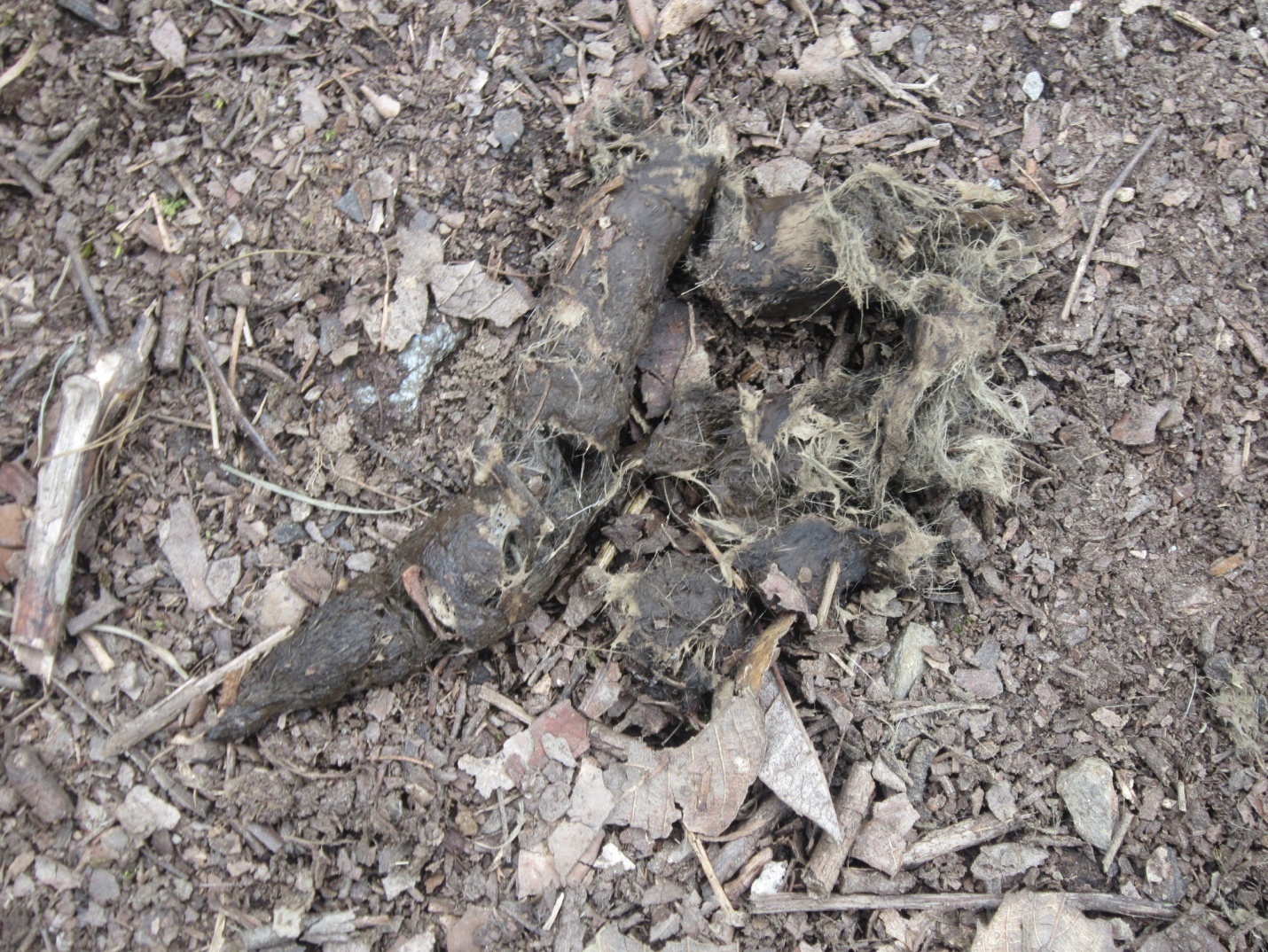


Scat of Asiatic Black Bear


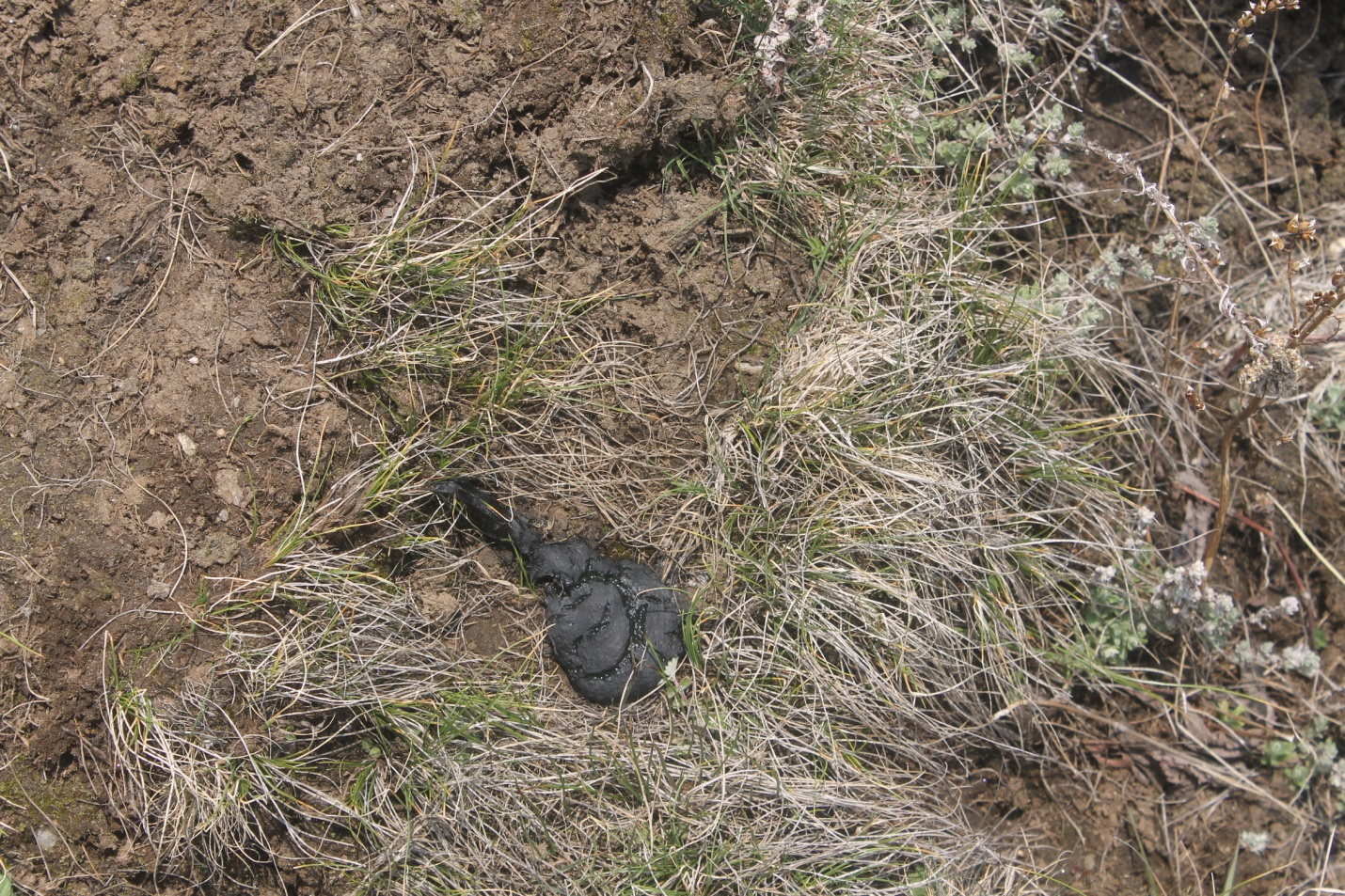

Supplement: Supplemental Information 1 [file peerj-11-16085-s001.docx]
